# The Makerspace Librarian’s Sourcebook

**DOI:** 10.5195/jmla.2017.337

**Published:** 2017-10-01

**Authors:** Elizabeth Connor

Few developments draw as much attention and excitement as spaces dedicated to makers, those people who imagine, design, innovate, and create. Despite the popularity of such spaces—especially in kindergarten through twelfth grade (K–12), public, and academic libraries—challenges remain about how to plan, fund, locate, implement, and sustain makerspace activities in an existing program that focuses on providing access to knowledge rather than creating it. Throughout this contributed work, the emphasis is on imagination, collaboration, and production rather than technology, but considerable attention is paid to specific tools for makers.

This guide is organized into three parts. The four chapters in part one focus on creating the space, connecting making to learning, developing the maker culture, and ensuring safety. Chapter 1, “How to Start a Library Makerspace,” includes equipment lists ranging in price from $500 to $50,000 and discussions of training, usage policies, and marketing. Chapter 2, “Pedagogy and Prototyping in Library Makerspaces,” emphasizes the value of dedicating library space toward active engagement, such as one sees in a makerspace environment, and how elements of prototyping and tinkering contribute to design thinking and deeper learning. Chapter 3, “Encouraging a Diverse Maker Culture,” addresses issues of accessibility and the importance of emphasizing “people and purpose rather than…things” (p. 52). Chapter 4, “Safety and Guidelines in the Library Makerspace,” stresses how training and supervision can foster a culture of safety that is needed to minimize the risk of injury.

Part Two includes eleven chapters devoted to specific tools such as 3D printers, Raspberry Pi, Arduino, wearable electronics, Google Cardboard, Legos, circuitry projects, milling machines, robotics, drones, and hackerspaces.

Part Three includes three chapters that consider the future of makerspaces, including using mobile units, sustaining user interest, identifying ongoing sources of funding, and using emerging technologies.

Each chapter includes pertinent references and resources. A comprehensive index helps with the usability of the work. This Facet Publishing title was originally published by the American Library Association. This book is recommended for both the curious and the committed who are looking for ways to justify and provide opportunities for makers in the realm of a library program.

